# Clinical and epidemiological factors causing longer SARS-CoV 2 viral shedding: the results from the CoviCamp cohort

**DOI:** 10.1007/s15010-023-02095-8

**Published:** 2023-09-13

**Authors:** Pierantonio Grimaldi, Antonio Russo, Mariantonietta Pisaturo, Paolo Maggi, Enrico Allegorico, Ivan Gentile, Vincenzo Sangiovanni, Annamaria Rossomando, Rossella Pacilio, Giosuele Calabria, Raffaella Pisapia, Canio Carriero, Alfonso Masullo, Elio Manzillo, Grazia Russo, Roberto Parrella, Giuseppina Dell’Aquila, Michele Gambardella, Antonio Ponticiello, Lorenzo Onorato, Nicola Coppola, CoviCam group, Caterina Monari, Caterina Sagnelli, Fabio Giuliano Numis, Carolina Rescigno, Angelo Salomone Megna, Vincenzo Esposito, Rodolfo Punzi, Francesco Maria Fusco, Giovanni Porta, Margherita Macera, Federica Calò, Angela Cascone, Gianfranca Stornaiuolo, Maria Stanzione, Paola Medusa, Carro Nicola, Andrea Dell’aquila, Simona Imbriani, Ricozzi Carmen, Klodian Gjeloshi, Roberta Astorri, Anna Maria Rossomando, Mariana Di Lorenzo, Giorgio Bosso, Claudia Serra, Ferdinando Dello Vicario, Valentina Minerva, Giulia De Angelis, Stefania De Pascalis, Salvatore Martini, Giovanni Di Caprio, Addolorata Masiello, Domenica Di Costanzo, Mariano Mazza, Vincenzo Bianco, Valeria Gentile, Antonio Riccardo Buonomo, Biagio Pinchera, Riccardo Scotto

**Affiliations:** 1 Department of Mental Health and Public Medicine, Section of Infectious Diseases, University of Campania Luigi Vanvitelli, Naples, Via L. Armanni 5, 80131 Naples, Italy; 2 Infectious Disease Unit, A. O. S Anna e S Sebastiano, Caserta, Italy; 3 Emergency Unit, PO Santa Maria delle Grazie, Pozzuoli, Italy; 4 Infectious Disease Unit, University Federico II, Naples, Italy; 5 Third Infectious Disease Unit, AORN dei Colli, P. O. Cotugno, Naples, Italy; 6 Infectious Diseases Unit and Gender Medicine, P. O. Cotugno, AORN dei Colli, Naples, Italy; 7 Hepatic Infectious Disease Unit, AORN dei Colli, PO Cotugno, Naples, Italy; 8 IX Infectious Disease Unit, AORN dei Coli, PO Cotugno, Naples, Italy; 9 First Infectious Disease Unit, AORN dei Coli, PO Cotugno, Naples, Italy; 10 Infectious Disease Unit, A.O. San Pio, PO Rummo, Benevento, Italy; 11 Infectious Disease Unit, A.O. San Giovanni di Dio e Ruggi D’Aragona, Salerno, Italy; 12 VIII Infectious Disease Unit, AORN dei Coli, PO Cotugno, Naples, Italy; 13 Infectious Disease Unit, Ospedale Maria S.S. Addolorata di Eboli, ASL Salerno, Salerno, Italy; 14 Respiratory Infectious Disease Unit, AORN dei Colli, PO Cotugno, Naples, Italy; 15 Infectious Disease Unit, AO Avellino, Avellino, Italy; 16 Infectious Disease Unit, PO S. Luca, Vallo della Lucania, ASL Salerno, Salerno, Italy; 17 Pneumology Unit, AORN Caserta, Caserta, Italy

**Keywords:** Time-to-negative swab, Viral shedding, COVID-19, SARS-CoV-2 infection, RT-PCR

## Abstract

**Introduction:**

The aim of this study was to investigate how long hospitalized patients stayed positive to the nasopharyngeal swab, and what demographic and clinical factors influence the time-to-negative swab.

**Methods:**

We enrolled in a multicenter, observational, retrospective study involving 17 COVID-19 units in eight cities of the Campania, southern Italy all patients hospitalized from March 2020 to May 2021 diagnosed with Severe Acute Respiratory Distress Syndrome-Coronavirus-2 (SARS-CoV-2) infection for whom time-to-negative swab was available.

**Results:**

963 patients were enrolled. We defined three groups considering time-to-negative swab: the first including patients with time-to-negative swab before the 26th day, the second including patients with time-to-negative swab from day 26 to day 39, and the third including patients with time-to-negative swab > 39 days. 721 (74.9%) patients belonged to the first group, 194 (20.1%) to the second, and 52 (5.4%) belonged to the third group. Belonging to group 2 and 3 seemed to be influenced by age (*p* value < 0.001), Charlson comorbidity index (*p* = 0.009), arterial hypertension (*p* = 0.02), cardiovascular disease (*p* = 0.017), or chronic kidney disease (CKD) (*p* = 0.001). The multivariable analysis confers a leading role to CKD, with an odds ratio of 2.3 as factor influencing belonging to the groups showing a longer time-to-negative swab. Patients with CKD and diabetes were more frequently in the third group.

**Discussion:**

Our analysis showed that CKD is a factor related to longer time-to-negative swab, probably because of immunosuppression related to this condition.

**Supplementary Information:**

The online version contains supplementary material available at 10.1007/s15010-023-02095-8.

## Introduction

To date, COronaVirus Disease-2019 (COVID-19) pandemic, caused by Severe Acute Respiratory Distress Syndrome-Coronavirus-2 (SARS-CoV-2) infection, has interested more than 700 hundred million cases and almost 7 million deaths worldwide [1].

Real-Time Polymerase Chain Reaction (RT-PCR) on nasopharyngeal swabs is considered the gold standard for the diagnosis of SARS-CoV-2 infection. Especially, during the first phases of the pandemic, it played a role in helping to end social isolation [2]. Soon afterward, antigenic swabs became central in controlling shelter-in-place isolation [3], with RT-PCR keeping a role where a certainty of non-contagiousness was required, e.g., in outpatient services for in-hospital admission.

In the literature, a few studies have focused on the issue of time-to-negative swab; nonetheless, some data on the positivity time span were possible to deduce from studies with different endpoints: this time ranged from 17 to 32 days in patients under 18 years old [4, 5], and from 14 to 27 days in adult patients [6–8]. Moreover, according to the recent studies conducted in the Omicron era, a prolonged time-to-negative swab, indicator of prolonged viral shedding, may be an element considered able to increase the risk of developing viral mutations [9]. The worry of the development of vaccine and therapy escape mutations has created the impulse to develop studies including time-to-negative swab or time-to-low viral load, still measured according to the high Ct levels in RT-PCR. Promising data show how early antiviral therapies can shorten the time-to-negative swab, potentially reducing mutation odds [10, 11]. From this point of view, identifying potential patients with longer than average time-to-negative swab may be an independent element in assessing who should receive early antiviral treatment; moreover, a prolonged time-to-negative swab was often a problem in the management of other co-pathologies where the paths dedicated to COronaVIrus Disease-2019 (COVID-19) were not identified.

The aims of the present study was to investigate positivity time spans in a large cohort of hospitalized patients with COVID-19 and the demographic and clinical factors influenced this time span, to determine which patients have a higher risk of long viral shedding.

## Materials and methods

### Study design and setting

We performed a multicenter, observational, retrospective study involving seventeen COVID-19 units in eight cities in the Campania region in southern Italy: Naples, Caserta, Salerno, Benevento, Avellino, Pozzuoli, Eboli, and Vallo della Lucania. All adult (≥ 18 years) patients, hospitalized with a diagnosis of SARS-CoV-2 infection confirmed by RT-PCR on a naso-oropharyngeal swab, from February 28th, 2020 to May 31st, 2021 at one of the centers participating in the study, were enrolled in the CoviCamp cohort [12–15]. No study protocol or guidelines regarding the criteria of hospitalization were shared among the centers involved in the study and the patients were hospitalized following the decision of the physicians of each center.

From the CoviCamp cohort, we included all patients for whom a time-to-negative swab was available. Exclusion criteria included minority age, lack of the data on the time-to-negative swab, and lack of informed consent; also, the patients who died during hospitalization were excluded.

The study was approved by the Ethics Committee of the University of Campania L. Vanvitelli, Naples (n°10877/2020). All procedures performed in this study were in accordance with the ethics standards of the institutional and/or national research committee and with the 1964 Helsinki declaration and its later amendments or comparable ethics standards. Informed consent was obtained from all participants included in the study.

This study was reported following the STROBE recommendations for an observational study (Supplementary Table 1).

### Variables and definitions

All demographic and clinical data of patients with SARS-CoV2 infection enrolled in the cohort were collected in an electronic database. From this database, we extrapolated the data for the present study.

The microbiological diagnosis of SARS-CoV-2 infection was defined as a positive RT-PCR test on a naso-oropharyngeal swab. All the units included used the same RT-PCR kit, Bosphore V3 (Anatolia Genework, Turkey, https://covid-19-diagnostics.jrc.ec.europa.eu/devices/detail/31). We considered the time-to-negative swab as the days from the first positive RT-PCR for SARS-CoV-2 to the first negative RT-PCR: the data were extrapolated from the regional database (https://sinfonia.regione.campania.it/preview/ecovid). Where available, we included the time from the last positive nasopharyngeal swab to the first negative nasopharyngeal swab for SARS-CoV-2.

We divided the patients enrolled according to the clinical outcome of COVID-19: patients with a mild outcome were those who did not require oxygen therapy, or performed oxygen therapy with a nasal cannula or venturi mask, hemodynamically stable patients, patients with a Glasgow Coma Scale greater than 9; patients with severe outcome were those who underwent oxygen therapy with high flow nasal cannula or non-invasive or invasive ventilation; non-hemodynamically stable patients, patients with a Glasgow Coma Scale less than 9.

### Statistical analysis

For the descriptive analysis, categorical variables were presented as absolute numbers and their relative frequencies. Continuous variables were summarized as mean and standard deviation or as median and interquartile range (Q1–Q3). We performed a comparison of patients with different time-to-negative swab (days) using Pearson chi-square or Fisher’s exact test for categorical variables and Student’s t test or Mann–Whitney test for continuous variables. Odds ratios were calculated using binomial logistic regression; these analyses were performed only for parameters clinically relevant and for those who resulted statistically significant at univariate analysis. We used the Benjamini–Hochberg (BH) procedure for controlling the false discovery rate (FDR) which entailed the correction of p values in the context of multiple comparisons. Analyses were performed by STATA [16].

## Results

**Fig. 1:**
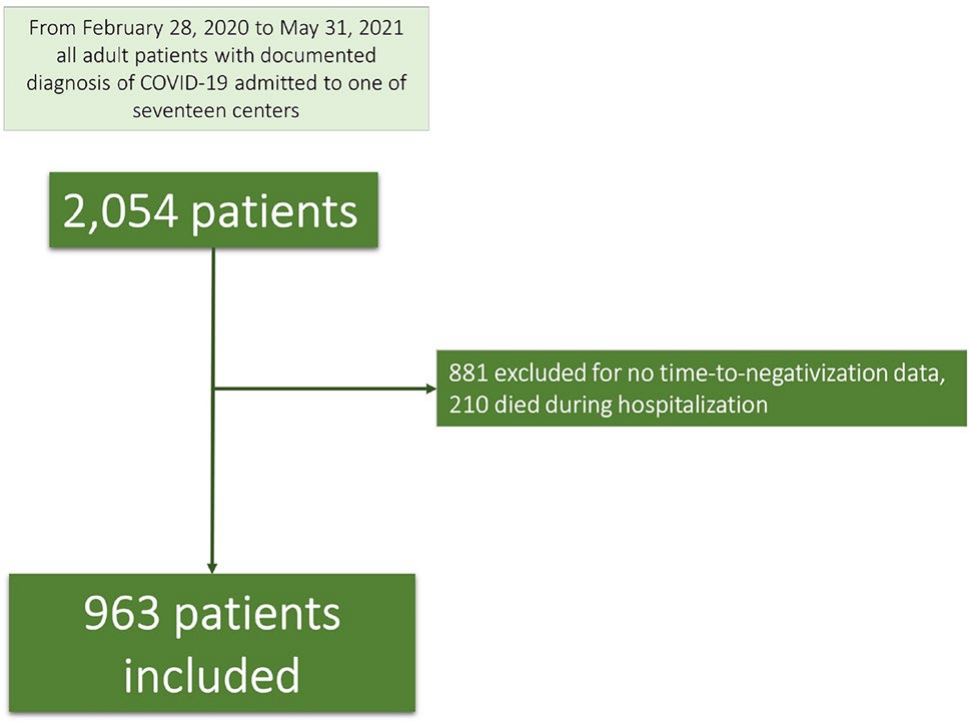
Flowchart of patients included in the study

During the study period, 2,054 patients with a diagnosis of SARS-CoV-2 infection, confirmed by a positive RT-PCR on a naso-oropharyngeal swab, were hospitalized in one of the centers participating in the study. We excluded 1,091 patients with insufficient data on swabs or those who died during hospitalization and, thus, 963 patients were enrolled for the present study (Fig. 1). The demographic and clinical characteristics of the 963 patients included in the study were similar to the 1,091 excluded (data not shown). Table 1 shows the demographic and clinical characteristics at admission and the clinical outcome of the 963 patients enrolled. Six hundred and eighteen (64.2%) subjects were male; median age was 61 years (Q1–Q3: 51–71 years). Overall, 60 patients (6.3%) had active cancer, and of these only 5 had a hematological malignancy. A severe outcome of COVID-19 was observed in 225 (23.5%) of the patients enrolled.

**Table 1:** Demographic, clinical, and laboratory data of the patients included

Variables included	
Patients included	963
Male, *n*° (%)	618 (64.2%)
Positivity time span, median (Q1–Q3)	19 (13–26)
Age, median (Q1–Q3)	61 (51–71)
Charlson comorbidity index, median (Q1–Q3)	2 (1–4)
Ongoing diseases	
Arterial hypertension, *n*° (%)	466 (48.6%)
Cardiovascular disease, *n*° (%)	249 (25.9%)
Chronic obstructive pulmonary disease, *n*° (%)	74 (7.7%)
Chronic kidney disease, *n*° (%)	64 (6.7%)
Cancer, *n*° (%)	60 (6.3%)
Chronic liver disease, *n*° (%)	35 (3.7%)
Diabetes, *n*° (%)	189 (19.7%)
Human immunodeficiency virus, *n*° (%)	8 (0.8%)
Dementia, *n*° (%)	31 (3.3%)
Symptoms at admission	
Fever, *n*° (%)	580 (60.7%)
Dyspnea, *n*° (%)	654 (68.5%)
Cough, *n*° (%)	315 (33.0%)
Fatigue, *n*° (%)	249 (26.2%)
Outcome of COVID-19	
Mild outcome	738 (76.5%)
Severe outcome	225 (23.5%)

The median time from the first positive to the first negative swab was 19 days (Q1–Q3: 13–26 days) (Table 1). To identify patients with an abnormally longer time-to-negative swab, representing long viral shedding, the patients who had the first negative swab before the 26th day (the 75th percentile) were included in Group A, those with a time to-negative-swab from 26 to 39 days (the 95th percentile) in Group B, and those with a time-to-negative swab > 39 days in Group C: the groups included 721, 194 and 52 patients, respectively. Since how often an SARS-CoV 2 positive patient would undergo a nasopharyngeal swab was an independent decision by the physician in care, we analyzed how long before the first negative swab the patients had had the previous swab: the median time was 5 (2–8) days in the first group, 6 (4–10) days in the second group, and 7 (4–13) days in the third group.

Table 2 shows the demographic, biochemical, and clinical data according to the three groups of patients. Median age was higher in groups B [64 years (55–72)] and C [67 years (57–77)] compared to Group A [60 years (50–70)] (*p < *0.0001). The Median Charlson Comorbidity Index was higher in group C [3 (2–5)] than in groups A and B (*p* = 0.009). Compared to the patients in group A, those in Groups B and C more frequently had arterial hypertension, cardiovascular diseases, and chronic kidney disease, differences all significant to the statistical analysis (Table 2). Linear-by-linear association was non-significant for arterial hypertension (*p* = 0.121), while it was significant for cardiovascular diseases and Chronic Kidney Diseases (CKD) (*p* = 0.002 and *p* = 0.003). Considering the clinical outcome of COVID-19, the prevalence of severe patients was higher with the increase in time-to-negative swab [(22% in group A, 26% in group B and 30.7% in group C) with a linear-by-linear association of 0.046].

**Table 2:** Demographic, clinical, and laboratory parameters of the patients grouped by time-to-negative swab

	A: < 26 days	B: ≥ 26. ≤ 39 days	C: ≥ 39 days	*p* value	Linear-by-linear association
Number of patients, *n*° (%)	721 (74.9%)	194 (20.1%)	52 (5.4%)		
Age, median (Q1–Q3)	60 (50–70)	64 (55–72)	67 (57–77)	** < 0.001**^**b**^	** < 0.001**
Males, *n*° (%)	460 (63.8%)	128 (67.3%)	30 (57.7%)	0.399^a^	0.936
Charlson Comorbidity index, median (Q1–Q3)	2 (1–4)	2 (1–4)	3 (2–5)	**0.009**^**c**^	**0.001**
Arterial hypertention, *n*° (%)	329 (45.8%)	114 (60%)	23 (44.2%)	**0.02**^a^	0.51
Cardiovascular disease, *n*° (%)	170 (23.6%)	60 (31.5%)	19 (36.5%)	**0.017**^a^	**0.005**
Chronic obstructive pulmonary disease, *n*° (%)	49 (6.9%)	19 (10%)	6 (11.5%)	0.194^a^	0.074
Chronic kidney disease, *n*° (%)	36 (5%)	23 (12.2%)	5 (9.6%)	**0.001**^a^	**0.002**
Cancer, *n*° (%)	41 (5.7%)	17 (8.9%)	2 (3.8%)	0.199^a^	0.537
Chronic liver disease, *n*° (%)	25 (3.5%)	10 (5.3%)	2 (3.8%)	0.642^a^	0.721
Diabetes, *n*° (%)	132 (18.3%)	43 (22.6%)	14 (26.9%)	0.168^a^	0.059
Dementia, *n*° (%)	22 (3.1%)	6 (3.2%)	3 (5.8%)	0.551^a^	0.4
Fever, *n*° (%)	430 (60%)	119 (63.3%)	31 (60.8%)	0.473^a^	0.967
Dispnea, *n*° (%)	484 (67.6%)	136 (72.3%)	34 (66.6%)	0.442^a^	0.505
Astenia, *n*° (%)	177 (24.8%)	53 (28.2%)	19 (37.2%)	0.116^a^	0.48
Cough, *n*° (%)	229 (32%)	66 (35.1%)	20 (39.2%)	0.455^a^	0.212
Patients with severe outcome of COVID-19 *n*° (%)	158 (22%)	51 (26%)	16 (30.7%)	0.232^a^	**0.046**
Days from last positive to first negative, median (Q1–Q3)	5 (2–8)	6 (4–10)	7 (4–13)	** < 0.001**^**c**^	** < 0.001**

*p* < 0.05 were written in Bold

^a^Chi-square test

^b^*t*-student test

^c^Mann–Whitney test

**Fig. 2:**
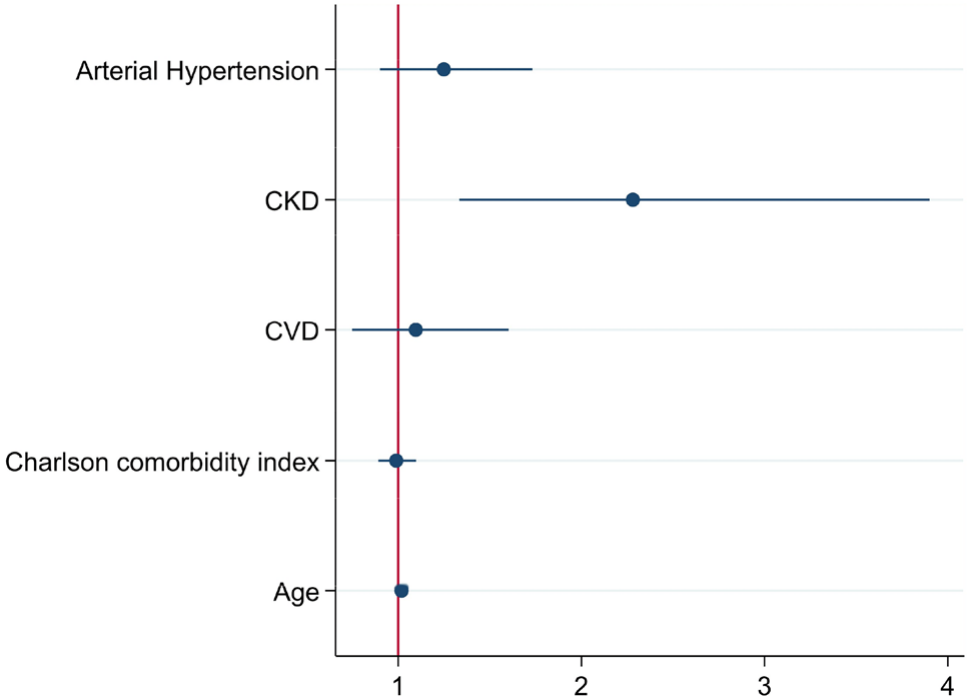
Forrest plot of predictors that increase time-to-negative swab

To identify independent factors associated with more prolonged viral shedding, we performed a multiple logistic regression on variables associated with belonging to groups with longer time-to-negative swab (Table 3 and Fig. 2): age and the presence of a history of CKD were the only independent factors associated with prolonged viral shedding: odds ratio (OR) 1.01 (95% Confidence Interval CI 1.00–1.03, *p* = 0.003) for age and OR 2.3 (95% CI 1.33–3.9, *p* = 0.025) for CKD. To control false discovery rate, we adjust p value with Benjamini–Hochberg method in multiple comparison test, after adjust p value was significant only for CKD in multivariable analysis (Table 2). To evaluate in the patients with CKD the role of diabetes in the prolonged time-to-negative swab, we defined two groups: the first including patients with diabetes and CKD, the second with CKD and no associated diabetes. Although between the two groups of patients, there was no statistical difference in time-to-negative swab [median 24 days (Q1–Q3: 17–33) vs 20 days (13–33) (*p* = 0.338)], 5 (16.7%) of the 30 patients with diabetes and CKD had a time-to-negative swab longer than 39 days compared to none of the 33 patients with CKD without diabetes (*p* = 0.045).

**Table 3:** Multivariable ordered logistic regression of parameters predicting the increase in time-to-negative-swab

	OR	95% Confidence interval	*p*	*p* adjust with BH procedure
Age	1.016	1.002–1.035	**0.025**	0.0625
Charlson Comorbidity Index	0.989	0.89–1.10	0.843	0.843
Arterial hypertension	1.249	0.90–1.73	0.182	0.303
Chronic kidney disease	2.281	1.33–3.90	**0.003**	**0.015**
Cardiovascular disease	1.096	0.75–1.60	0.636	0.795

*p* < 0.05 were written in Bold

## Discussion

In this multicenter observational retrospective study, our aim was to find demographic and clinical factors that may predict persistency of positive nasopharyngeal specimen to SARS-CoV-2. In our population of 963 patients, we identified a median time-to-negative swab of 19 days (Q1–Q3: 13–26 days) and in about 5% of the population very prolonged SARS-CoV-2 shedding (more than 39 days after the first positive swab). Moreover, the prevalence of patients with severe COVID-19 increased with the increase in time-to-negative swab.

Persistence of SARS-CoV-2 is a current issue considering its impact and the poor scientific evidence on the management of this condition. Studies have underlined the impact of persistent viral shedding, especially in immunocompromised populations: prolonged viral shedding was associated with higher infectivity, the accumulation of mutations, immune escape, and poor prognosis because of the consequent impossibility to give treatments for the ongoing disease [9, 17]; furthermore, it was associated with in-hospital delirium (OR 2.43; 95% CI 1.42, 4.29) [18]. The scientific community is aware of this issue and is currently trying to improve the management of patients with prolonged viral shedding. For example, case reports and studies have highlighted how the combination of different antiviral drugs commonly used in early treatment could be effective in inducing virus clearance, especially in patients with a hematological malignancy [19–22]

Previous studies have evaluated the predictive factors for prolonged viral persistence. The factors highlighted as prognostic were both demographic factors such as age and sex, but also clinical factors such as the presence of hypertension and pharmacological treatments [23–25]. Differently from our study, these studies included patients who were asymptomatic or had mild symptoms [24, 25] and one included only 113 patients in early 2020 [23]. Mostly prolonged viral shedding was observed in immunocompromised populations [26] especially in hematological malignancies, with an average negativization time of about 4 weeks [27].

In the present study on 963 hospitalized COVID-19 patients, the variable independently associated with a longer viral shedding was the presence of CKD (OR 2.3, 1.33–3.9). Moreover, the patients with CKD and diabetes were more likely to have a longer time-to-negative swab, frequently more than 39 days.

The association of CKD as a factor that can increase time-to-negative swab, to our knowledge, is a new and relevant factor. In fact, previously, several studies showed that patients with CKD had higher mortality and worse outcome compared with patients without [28–30], but, to our knowledge, no studies showed the impact of CKD on the time-to-negative swab. The persistence of SARS-CoV-2 in patients with CKD observed in the present study could be related to an alteration in the immune system due to renal impairment [31]. In fact, in patients with CKD, the increase in pro-inflammatory cytokines, the increase in acute phase protein, and the dysfunction of phagocytes B- and T-cells can lead to systemic inflammation and acquired immunodepression, which accounts for infectious complications [31], including a prolonged SARS-CoV-2 shedding. In addition, our data showed that patients with diabetes and CKD were more likely to have a time-to-negative swab longer than 39 days compared to patients without diabetes with CKD. These data may be due to the impact of diabetes on inflammatory pathways [32]: diabetes leads to a dysregulation of immune pathways such as an alteration in signaling mediated by Toll-like receptors, an alteration in NLR family Pyrin domain containing 3 (NLRP3) inflammasome with the activation of a pro-inflammatory cascade of Il-1β and IL-18 [32], probably increasing immune alteration.

Our study shows several limitations. First, its retrospective design. Second, we had very low prevalence of patients with a hematological malignancy and given the small number may not have shown the correlation with the time-to-negative swab. Third, swabs were performed without a pre-fixed timing, so each test result depicted a result from a random sample at a random point in time [33]; furthermore, there was a small but significant difference between the groups considering the time from last positive test and the first negative; however, it is usual in clinical practice to delay the SARS-CoV-2 nasopharyngeal test in patients who are positive for longer. Fourth, due to the historical period of our study, our cohort of patients did not include subjects who were vaccinated against SARS-CoV-2 and/or early treatments (antivirals or monoclonal antibodies), nor the impact of different variants. Fifth, we excluded patients who died during hospitalization. Sixth, it has to be taken into account a certain degree if false positive test risk in patients with long viral shedding, as for example, due to the detection of inactive viral particles [34]. Seventh, because of the absence of data, no longitudinal survival model was be performed in our study. On the other hand, the strengths of our study are the multicenter design, the sample size of the population, and the heterogeneity of age and multi-co-morbidities, which may be considered representative in the setting of hospitalized COVID-19 patients.

## Conclusion

In conclusions, our study estimated that about 5% of hospitalized COVID-19 patients were SARS-CoV-2 positive at nasopharyngeal swab for at least 39 days after the first positive. Moreover, the patients with CKD and diabetes were more likely to have a time-to-negative swab of more than 39 days, probably due to immune system alterations related to this condition.

### Supplementary Information

Below is the link to the electronic supplementary material.

Supplementary file1 (DOCX 19 KB)

## Data Availability

The data that support the findings of this study are available from the corresponding author, Coppola Nicola, upon reasonable request.
